# Simple Shapes Elicit Different Emotional Responses in Children with Autism Spectrum Disorder and Neurotypical Children and Adults

**DOI:** 10.3389/fpsyg.2017.00091

**Published:** 2017-01-30

**Authors:** Laurine Belin, Laurence Henry, Mélanie Destays, Martine Hausberger, Marine Grandgeorge

**Affiliations:** ^1^Laboratoire d’Éthologie Animale et Humaine EthoS–UMR-CNRS 6552, Université de Rennes 1Rennes, France; ^2^CNRS, Laboratoire d’Éthologie Animale et Humaine EthoS–UMR-CNRS 6552, Université de Rennes 1Rennes, France; ^3^Laboratoire d’Éthologie Animale et Humaine EthoS–UMR-CNRS 6552, Station Biologique de Paimpont, Université de Rennes 1Paimpont, France

**Keywords:** humans, autism spectrum disorder, emotional response, simple shapes, behavior

## Abstract

According to the literature, simple shapes induce emotional responses. Current evaluations suggest that humans consider angular shapes as “bad” and curvilinear forms as “good,” but no behavioral data are available to support this hypothesis. Atypical development, such as autism spectrum disorder (ASD), could modify humans’ perception of visual stimuli and thereby their emotional effect. This study assessed the effects of simple stimuli (i.e., jagged edges shape, disk, star, spiral, eye-like shape, and head character) on the emotional responses of different groups of humans. First, we assessed the effects of a looming movement on neurotypical adults’ emotional responses. Second, we assessed the effects of atypical development on emotional responses by comparing the reactions of neurotypical children and of children with ASD. We used different methodological approaches: self-evaluation through questionnaires and direct observation of participants’ behavior. We found that (1) neurotypical adults tended to perceive looming stimuli negatively as they associated more negative feelings with them although few behavioral responses could be evidenced and (2) the emotional responses of neurotypical children and of children with ASD differed significantly. Neurotypical children perceived the spiral stimulus positively, i.e., a curvilinear shape, whereas children with ASD perceived the jagged edges stimulus positively, i.e., an angular shape. Although neurotypical children and children with ASD presented some behavioral responses in common, children with ASD smiled and vocalized more than did neurotypical children during stimuli presentations. We discuss our results in relation to the literature on humans’ perception of simple shapes and we stress the importance of studying behavioral components for visual cognition research.

## Introduction

Rapid perception and precise recognition of relevant stimuli are important for individual survival. Consequently, preferential processing is paid to potential rewarding and threatening stimuli (e.g., Gable and Harmon-Jones, 2008). Moreover, each individual has its own perceptual world meaning that perception differs among species and among individuals according to their sensory, evolutionary, and developmental history (von Uexküll, 1934). However, despite different perceptual “equipment,” various species may share similar perceptual and emotional responses to particular visual stimuli. For example, both human and non-human primates present an attentional bias toward general biological threat-relevant stimuli, such as threatening conspecific faces (Kawai et al., 2015). Moreover, eyespots are relevant stimuli for numerous species (e.g., on butterfly wings, fish fins...), as they play an important antipredator role (e.g., inducing avoidance, freezing; Jones, 1980; Inglis et al., 1983; Stevens et al., 2008).

Biomimetic (i.e., imitating natural) stimuli may induce emotional responses in humans (e.g., preference; behavioral reactions; Globish et al., 1999; Öhman et al., 2001; Tipples et al., 2002). Thus, human facial configurations induce various emotional responses. Whereas angular human faces convey threatening information (e.g., Aronoff et al., 1988), juvenile human faces convey positive information and increase bonding and attachment (Hildebrandt and Fitzgerald, 1983). More generally, human adults with juvenile features (e.g., round face, large round eyes, large pupils: “baby face”) are preferred and perceived as pleasant, reflecting a more warm-hearted, honest, and kinder personality (Lorenz, 1943; Berry and McArthur, 1985; Zebrowitz and Montepare, 1992; Zebrowitz, 1997). Similar ratings are reported for juvenile-type animal faces (e.g., Borgi et al., 2014; Borgi and Cirulli, 2016). Cuteness can increase both humans’ and animals’ caring attention (Sherman et al., 2009; Sherman and Haidt, 2011). Conversely, threat-relevant stimuli capture attention and induce unconscious startle responses (Öhman, 2005). Most reports deal with recurrent threat-relevant stimuli such as snakes or spiders (e.g., Öhman and Mineka, 2001; Coelho and Purkis, 2009).

Preferences or dislikes for, and behavioral responses to, biomimetic stimuli are not universal and depend on cognitive processes. For example, humans with atypical development such as people with autism spectrum disorder (ASD) present sensory alterations inducing them to be attracted or repulsed by visual stimuli (Ben-Sasson et al., 2009) such as rolling objects (Gepner et al., 2010). Grandin (2009), an adult with ASD, explained that “as a child, [her] favorite repetitive behavior was dribbling sand through her hands over and over. The reason […] was [her] fascination with the shapes and reflections off of every tiny grain.” Children with ASD may express unusual fears of, for instance, vacuum cleaners, tornadoes, shadows, or plants (Mayes et al., 2013). These stimuli can elicit aberrant behavioral responses, e.g., tantrums and crying.

As Bar and Neta (2006) reported, the emotional valence of stimuli is induced by their semantic meaning as well as by simple level properties of the environment (e.g., shape, symmetry, prototypicality, contrast, complexity, or perceptual fluency; Reber et al., 2004). These properties affect humans’ preferences, judgments, behavioral responses, and decisions (Palmer et al., 2013). For example, looming movements induce human infants to retreat (Ball and Tronick, 1971). Various animal species also respond to looming movements with similar negative reactions (e.g., Burger and Gochfeld, 1981; Hassenstein and Hustert, 1999; Carlile et al., 2006). Moreover, threat-relevant stimuli presented with a looming movement capture humans’ attention (Hillstrom and Yantis, 1994; Franconeri and Simons, 2003).

Simple shapes also induce emotional responses by humans. Ratings reveal that straight lines and angular shapes (especially a downward V) are considered “bad” and circles and curvilinear shapes are considered “good” (e.g., Aronoff et al., 1992; Larson et al., 2007; Watson et al., 2012; Chen et al., 2015). This preference for curvilinear shapes seems to be present early during development, before language is acquired (Amir et al., 2011). Moreover, authors report preference relationships between simple properties (i.e., colors and shapes; Chen et al., 2015). Interestingly, simple shapes activate different neural networks, in particular shapes with a downward V activate neurotypical humans’ neural circuitry linked to threat detection (Larson et al., 2009). To our knowledge, similar data concerning simple shapes are not available for people with ASD, with the exception of one report showing that children and infants with ASD prefer geometrical stimuli to social stimuli (Shi et al., 2015; Chavis, 2016). Nevertheless, researchers showed that people with ASD have difficulties perceiving simple level properties of stimuli such as movement (especially when complex or rapid: Bertone et al., 2003; Gepner and Féron, 2009) or color (Franklin et al., 2008).

All previous research investigating effects of simple level properties of stimuli on emotional responses used questionnaires and some included neurophysiological investigations (e.g., eye tracking, EEG). To our knowledge, direct observations applying ethological methods (Altmann, 1974) have never been used. However, this type of observation can yield information concerning behavioral reactions and the underlying mechanisms involved whereas questionnaires explore human attitudes, feelings, representations, and/or preferences. As Kingstone et al. (2008) stressed, human cognition must be investigated at least at two levels (i.e., personal and sub-personal) and this can be done in particular by direct observation. Combining several methods yields more complete answers to this question (i.e., effects of simple level properties of stimuli on humans’ emotional responses).

Based on these previous studies, we hypothesized that simple shapes would induce emotional responses, i.e., preferences and behavioral reactions, and that atypical development (e.g., ASD) would influence emotional perception. To test this hypothesis, we presented a set of looming and static simple stimuli with either an expected positive valence (i.e., curvilinear shaped stimuli) or negative valence (i.e., angular shaped stimuli) (e.g., Larson et al., 2007, 2012) to different groups of humans. We used two methodological approaches: self-evaluation of subjective perception (feelings) and preferences using questionnaires and direct observations of behavioral reactions (Altmann, 1974). First, we assessed the effects of a looming movement on neurotypical adults’ emotional responses. Second, we assessed the effects of an atypical development on emotional responses to looming stimuli by comparing responses of neurotypical children and of children with ASD.

## Materials and Methods

This investigation was conducted according to the principles expressed in the Helsinki Declaration. All experimental protocols were consistent with the Guide for Experimentation with Humans, and were approved by the Institutional Ethic Committee of Rennes, France (O15/01-003). In accordance with the ethics committee and prior to their inclusion, adult participants were fully informed and gave their consent, and parents gave an informed consent to allow their child to participate in the experiment.

Details were given after in each experiment section.

### Participants

This study included 56 subjects. All of the child and adult participants had normal or corrected-to-normal vision. The present research was non-invasive and did not involve pharmacological interventions.

### Design

**FIGURE 1:**
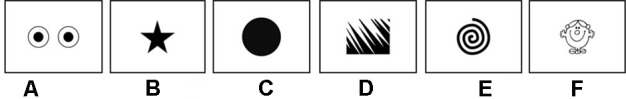
**Visual stimuli: (A)** eye-like shape; **(B)** star; **(C)** disk; **(D)** jagged edges shape; **(E)** spiral, and **(F)** head character. Experiment 1: the first five stimuli **(A–E)** were presented to neurotypical (NT) adults in a static version and a looming version. Experiment 2: all six stimuli **(A–F)** were presented to NT children and to children with autism spectrum disorder (ASD) in a looming version. Author credits: Roger Hargreaves.

The stimuli, presented on PowerPoint slides, were a black eye-like shape, a star, a spiral, jagged edges shape, a disc, and a head character (**Figure 1**). The background was white. They were presented on a monitor placed in an isolated room. Participants were instructed to wait and watch the screen. At the end of the experiment, they had to fill in a questionnaire.

### Data collection

#### Questionnaire on Preferences and Feelings

This questionnaire invited the participants to indicate the picture they preferred and the one they disliked the most among all those perceived during the experiment. Then participants had to attribute one of the three following qualifying terms to each stimulus: pleasure, indifference, or dislike. To help the children, (1) smileys were used to illustrate feelings and (2) the experimenter read the questions and made sure that they had been understood.

#### Observations for Behavioral Data

During both experiments, the behavior of each participant was video-recorded (Canon HG21). Ethological methods of data sampling were used to collect behavioral data, here, *one-zero sampling* (Altmann, 1974) during 10 s before the stimulus appeared and during the 10 s it was projected.

Behavioral data were analyzed by two experimenters. These experimenters possessed a strong background in ethology and so were familiar with behavioral analyses for both humans and animals. They were formed until inter-rater reliability was more than 95%. Then each of them rated a part of the video-recorded behaviors. The videos were analyzed with Kinovea software 0.8.11 that enables frame by frame analysis.

The behavioral items recorded were noted in terms of presence/absence:

(1) Redirected activities (Troisi, 2002): head, arm, hand, or legs movements directed away from the principal target, i.e., toward the screen.
(2) Self-centered activities: all behaviors centered on self, e.g., scratching.
(3) Spontaneous vocalizations: words uttered or exclamations without answering a question or request.
(4) Four types of facial movements: raising eyebrows, frowning, pinching mouth and smiling (Ekman and Rosenberg, 1997). Only simple data were collected for a first behavioral approach.
(5) Postural changes: sitting position in relation to the table, i.e., either upright (90°), leaning forward (<90°), or leaning backward (>90°).

### Statistical Analyses

As our data did not fit a normal distribution, we applied non-parametric statistical tests (Siegel and Castellan, 1988). McNemar tests were used to compare data for static and looming stimuli according to (1) the responses for the preferred and disliked stimulus, (2) the responses for the emotional feeling related to each stimulus, (3) modifications of all the behavioral items recorded between the two periods (i.e., 10 s before a stimulus appeared and during all the 10 s the stimulus was displayed). McNemar, Cochran, and Fisher tests were used to assess the effects of atypical development (i.e., ASD). Intra- and intergroup comparisons (ASD versus NT) were performed according to (1) the responses for the preferred and disliked stimuli, (2) the responses for the emotional feeling related to each stimulus, (3) modifications of all the behavioral items between the two periods (i.e., 10 s before a stimulus appeared and during the 10 s of stimulus display). Bonferroni corrections were applied when necessary.

## Experiment 1: Effects of Looming Movements of Stimuli on NT Adults’ Emotional Responses

### Specific Methods

#### Participants

The subjects were 26 neurotypical (NT; 13 women and 13 men), 19–25 years old (*M*: 22; *SE*: 0.25), French adult students at the University of Rennes 1 (France). They were all voluntary, recruited following advertisements. To be eligible, they had to be between 18 and 25 years old. We excluded individuals (1) with genetic or neurological (or suspected) abnormalities and/or (2) with non-corrected vision.

#### Apparatus, Stimuli, and Specific Procedure

The stimuli were presented on a 48 cm (diagonal) Hyundai X93Wd monitor at 1440 × 900 resolutions, refreshed at 60 Hz. Adults sat with their eyes approximately 50 cm from the screen. Each adult was presented either the static or the looming stimuli (A–E) in a random order (**Figure 1**). The looming version was the expansion of the stimulus at a frequency of 0.5 Hz. Each projection of a stimulus (static or looming) lasted 10 s. A white slide was projected between two stimuli and lasted randomly for 30, 40, or 50 s.

Non-parametric ANOVAs were computed to compare valence ratings across stimuli in addition to McNemar and Cochran tests (numerical values were applied to subjects’ valence answers: negative feeling = -1; neutral feeling = 0; positive feeling = +1).

### Results

#### Preferences and Feelings

**FIGURE 2:**
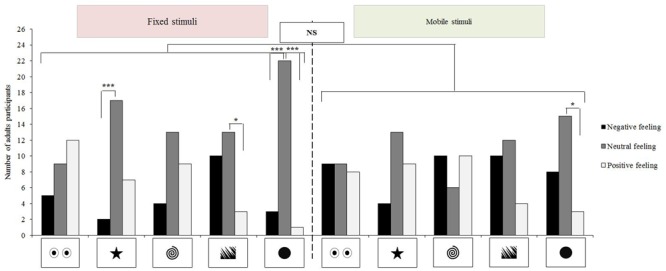
**Association stimulus-feelings for NT adults (*n* = 26).** McNemar and Cochran tests, ^∗∗∗^*p* < 0.001; ^∗^*p* < 0.05, NS, not significant.

The participants preferred the looming disc to all the other stimuli (McNemar test: *n* = 26; *X*^2^= 4.17, *p* = 0.041). No other significant differences could be evidenced either for other preferred stimulus (0.00 ≤*X*^2^≤ 0.75, *p* ≥ 0.387) or for disliked pictures (0.00 ≤*X*^2^≤ 0.80, *p* ≥ 0.371). Non-parametric ANOVAs could not evidence any significant effects of static or looming stimuli on adults’ feelings (non-parametric ANOVAs: static stimuli: *p* ≥ 0.080; looming stimuli: *p* ≥ 0.220). Subjects overall expressed more negative feelings for the looming stimuli although this could not be tested statistically, due to the small sample size (*N* = 5). The other feelings (i.e., indifference and pleasure) were less impacted by looming movements (*p* > 0.1; **Figure 2**). Comparisons at stimulus level did not evidence any effects of looming movements on feelings (McNemar tests: *dislike*: *n* = 26; 0.00 ≤*X*^2^≤ 3.13, *p* ≥ 0.077; *indifference*: *n* = 26; 0.00 ≤*X*^2^≤ 3.27, *p* ≥ 0.071; *pleasure*: *n* = 26; 0.00 ≤*X*^2^≤ 1.50, *p* ≥ 0.221). Significantly more adults reported neutral feelings in the presence of the static star, static jagged edges (respectively; Cochran test: *Q* = 11.84, *p* = 0.002; *Q* = 6.25, *p* = 0.036; **Figure 2**) and static or mobile disk stimuli (static: *Q* ≤ 19.17, *p* < 0.001; mobile: *Q* = 8.00, *p* = 0.015; **Figure 2**).

#### Behavioral Reactions

54% of the NT adults modified their postural during a stimulus display, but the looming stimuli did not impact postural changes (McNemar test: *n* = 26; 0.00 ≤ *X*^2^≤ 0.80, *p* ≥ 0.371). Sixty-nine percentage of the adults displayed at least one redirected activity 10 s before the stimulus appeared and 58% did during the stimulus display (*X*^2^= 0.90, *p* = 0.343). Thirty-eight percentage of the NT adults displayed at least one self-centered activity before the stimulus appeared and 35% did during the stimulus display (*X*^2^= 0.00, *p* = 1.000). Looming movements did not induce significant changes in these activities (*redirected activities*: 0.00 ≤*X*^2^≤ 3.20, *p* ≥ 0.074; *self-centered activities*: 0.00 ≤*X*^2^≤ 1.33, *p* ≥ 0.248). The display of the stimuli induced some changes in the adults’ behavior. Before the stimuli appeared, none of the NT adults frowned while 23% did during display (*before vs. during, X*^2^= 4.17, *p* = 0.041). No other significant changes were observed (*before*: 0% raised their eyebrows, 50% pinched their mouth, and 8% smiled; versus *during*: 8% raised their eyebrows, 35% pinched their mouth, and 23% smiled; Khi-square all *p* > 0.05). However, no significant changes between periods of any of the facial movements could be attributed to the looming movement (*all facial movements*: 0.00 ≤*X*^2^≤ 0.50, *p* ≥ 0.480). No adult vocalized spontaneously either before or during the stimuli displays.

## Experiment 2: Effects of Atypical Development on NT and ASD Children’s Emotional Responses to Looming Stimuli

### Specific Methods

#### Participants

The subjects were 28 French children. Fourteen to fifteen years old (*M* = 8.4; *SE* = 0.9) children with ASD (two girls and 12 boys) came from the “Centre de Ressources sur l’Autisme de Bretagne,” Bohars, France. They were matched for chronological age with 14 4–15 years old (*M* = 8.4; *SE* = 0.8) neurotypical children (NT; two girls and 12 boys; Mann–Whitney test, *n*_1_ = 14, *n*_2_ = 14, *U* = 97.5, *p* = 1.000). The NT children attended school regularly; none met any diagnostic criteria for ASD or other pervasive developmental disorders. Before the experiment and based on direct clinical observation of children with ASD by independent child psychiatrists, a diagnosis of ASD was made according to DSM-IV (American Psychiatric Association [APA], 1994) as well as ICD-10 (World Health Organization, 1994) criteria and included an assessment with the ADI-R (Lord et al., 1994). The ADI-R scale evaluates the degree of disability of subjects in three major domains of autistic impairment: reciprocal social interactions, verbal and non-verbal communication, stereotyped behavior, and restricted interests. ASD symptoms were assessed with the ADI-R items included in the algorithm: total social interaction score (15 items with a threshold of 10), total verbal/non-verbal communication score (13 items and nine items, respectively, with thresholds of 8 and 7), and total stereotype score (eight items: threshold of 3). These scores are reported in **Table 1**. Despite the fact that not all the children fill the three criteria of the ADI-R scale (i.e., six children on 14) they were diagnosed autistic with the results of the IQ tests and clinical interview. Using different psychological tests (e.g., WISC 4 and K.ABC), we identified that no child had intellectual disability (IQ score: *M* = 89.4; *SD* = 14.7; min–max = 70–116).

**Table 1:** Characteristics of children with autism spectrum disorder (ASD; *N* = 14) according to the ADI-R.

	Mean ±*SD*	Min–Max
Social interaction	17.7 ± 5.0	9–24
Verbal communication	14.3 ± 6.7	6–25
Non-verbal communication	7.2 ± 4.6	1–14
Stereotypes	6.7 ± 2.8	0–12

#### Apparatus, Stimuli, and Specific Procedure

The stimuli were presented on a 35.6 cm (diagonal) HP EliteBook 8470p monitor at 1600 × 900 resolutions, refreshed at 60 Hz placed on a table in the child’s home. Children sat with their eyes approximately 50 cm from the screen. The children were presented only the looming versions of the stimuli (A–F) and in a fixed order (**Figure 1**). The looming versions were the expansion of the stimuli at a frequency of 0.5 Hz. Each projection of a stimulus lasted 10 s. A white slide was projected for 2 min between two stimuli.

The head stimulus from “Mr Men and Little Miss” was added to give a social dimension to the test as this element presents difficulties for children with ASD (Volkmar and Pauls, 2003).

Given the wide age range of the child participants (4–15 years) we divided them into two age classes (under or over 8 years old) to analyze their feeling and redirected activity data.

### Results for NT Children

Neurotypical children’s feelings did not vary significantly with age class (i.e., under *vs.* over 8 years old; Fisher test: *N* = 14; *p* ≥ 0.055) except for the jagged edges stimulus. Seventy-five of the young child participants expressed positive feelings in presence of this stimulus compared to none of child over 8 years old (*p* = 0.010). This class of NT children (i.e., over 8 years old) expressed more neutral feelings in presence of the jagged edges stimulus than younger children (*p* = 0.026). Redirected activities performed by NT children did not vary significantly with age class (Fisher test: *N* = 14; *p* ≥ 0.209). This absence of significant differences between age classes allowed us to pool all data for NT children.

#### Preferences and Feelings

**FIGURE 3:**
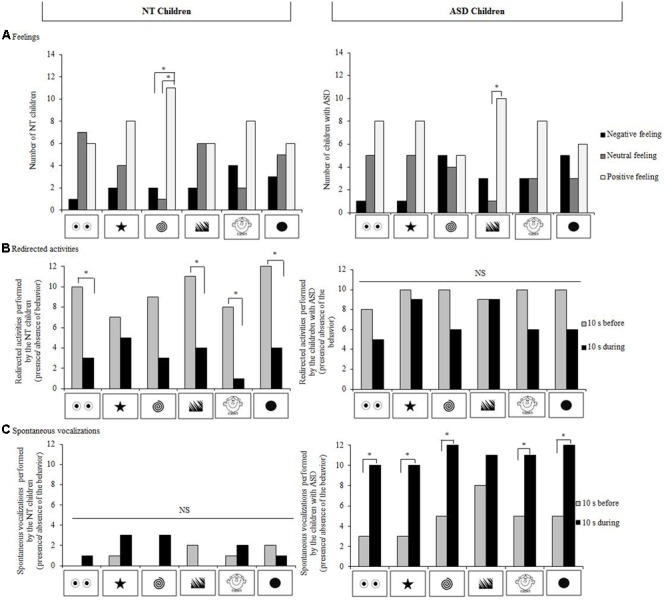
**Feelings and behavioral reactions of our NT children and children with ASD (*N* = 14 for each group). (A)** Types of feelings expressed for the different stimuli; **(B)** Redirected activities before a stimulus appeared and during the stimulus display, and **(C)** Spontaneous vocalizations before the stimulus appeared and during stimulus display. Cochran and McNemar tests; ^∗^*p* < 0.05, NS, not significant.

When data for all the stimuli were taken into account, no particular preference (Cochran test: *N* = 14; 0.00 ≤*Q* ≤ 4.00, *p* ≥ 0.683) or dislike for a particular stimulus could be evidenced (0.00 ≤*Q* ≤ 2.67, *p* = 1.000). Overall, NT children expressed positive feelings toward the stimuli displayed during the experiment and NT children expressed more positive than neutral or negative feelings in presence of the spiral (*positive vs. neutral*: *Q* = 6.23, *p* = 0.039; *positive vs negative*: *Q* = 8.33, *p* = 0.012; **Figure 3A**).

#### Behavioral Reactions

Globally, 50% of the NT children modified their posture during a stimulus presentation. Nevertheless, no particular stimulus induced significantly more postural changes than the other stimuli (0.00 ≤*Q* ≤ 5.20, *p* ≥ 0.074). All the NT children performed at least one redirected activity before a stimulus appeared and 57% did during its presentation (*X*^2^= 4.17, *p* = 0.041). NT children decreased their redirected activities in all cases and significantly so for the eye-like shape, the jagged edges, the head, and the disk stimuli (McNemar test: *n* = 14; 4.00 ≤*X*^2^≤ 6.13, *p* ≥ 0.013) but not for the star (*X*^2^= 0.13, *p* = 0.724) or the spiral (*X*^2^ = 3.13, *p* = 0.077; **Figure 3B**). Eighty-six percentage of the children performed at least one self-centered activity and 36% did during stimuli display (*X*^2^ = 5.14, *p* = 0.023). Numbers of self-centered activities decreased during presentations of stimuli in all cases but this was statistically significant only during the jagged edges display (*X*^2^ = 4.17, *p* = 0.041). Before the stimuli appeared, none of the NT children raised their eyebrows whereas 64% did during a display (*before vs. during, X*^2^= 7.11, *p* = 0.008). No other significant differences were observed (*before*: 0% frowned their eyebrows, 43% pinched their mouth, and 22% smiled; *versus during*: 22% frowned their eyebrows, 64% pinched their mouth, and 57% smiled; Khi-square all: *p* > 0.05). Whatever the facial movement, NT children’s facial movements did not change significantly despite a trend to pinch their mouth (2.22 ≤*Q* ≤ 10.45, *p* ≥ 0.063). Fourteen percentage of the NT children uttered at least one vocalization before a stimulus appeared and 36% did during its presentation (*X*^2^= 1.33, *p* = 0.248). As for the other behaviors, none of the stimuli induced significant changes of spontaneous vocalizations (0.00 ≤*X*^2^≤ 1.33, *p* ≥ 0.248).

### Results of ASD Children

As for the NT children, age class (i.e., under *vs* over 8 years old) had no significant effect on the feelings of children with ASD (Fisher test: *N* = 14; *p* ≥ 0.138) and redirected activities (*p* ≥ 0.138). So for the rest of analysis we could pool data for all the children with ASD.

#### Preferences and Feelings

As the NT children, children with ASD showed no particular preference (0.00 ≤ *Q* ≤ 1.80, *p* = 1.000) or dislike for a particular stimulus (0.00 ≤*Q* ≤ 1.80, *p* = 1.000). Overall, the children expressed predominantly positive feelings toward the stimuli, especially for the jagged edges (positive *versus* neutral feeling; *Q* = 7.36, *p* = 0.021); no significant associations could be evidenced for the other stimuli (0.00 ≤*Q* ≤ 5.44, *p* ≥ 0.060; **Figure 3A**).

#### Behavioral Reactions

Overall, 86% of the children with ASD made a postural change during displays, but no stimulus in particular induced significant postural changes (0.25 ≤*Q* ≤ 4.00, *p* ≥ 0.135). Before the stimuli appeared, all the children with ASD performed at least one redirected activity and 86% performed at least one self-centered activity. During the display, 79% performed a redirected activity *(before vs. during, X*^2^ = 1.33, *p* = 0.248) and 36% a self-centered activity (*X*^2^= 4.00, *p* = 0.046). The number of children with ASD displaying redirected and self-centered activities tended to decrease when a stimulus was presented (0.00 ≤*X*^2^≤ 2.25, *p* ≥ 0.134; **Figure 3B**) and it decreased significantly in the presence of the head character stimulus (*X*^2^= 4.17, *p* = 0.041). Before the stimuli appeared, none of the ASD children raised their eyebrows whereas 72% did during display (*before vs. during, X*^2^= 7.11, *p* = 0.008). Before the stimuli appeared, 29% of the children with ASD smiled whereas 79% did during display (*before vs. during, X*^2^= 5.14, *p* = 0.023). No other significant results could be evidenced (*before*: 0% frowned their eyebrows and 36% pinched their mouth; *versus during*: 29% frowned their eyebrows and 50% pinched their mouth; both Khi-square tests: *p* > 0.05). Children with ASD raised their eyebrows more during the presentation of the stimuli (4.17 ≤*X*^2^≤ 5.14, *p* ≥ 0.023) except for the eye-like shape stimulus (*X*^2^= 2.25, *p* = 0.134) and the star stimulus (*X*^2^= 2.25, *p* = 0.134). More children with ASD smiled for the head character stimulus (*before versus during*; *X*^2^= 4.17, *p* = 0.041). None of the other facial movements were influenced by a stimulus display (*frowning*: 0.00 ≤*X*^2^ ≤ 1.33, *p* = 0.248; *pinching lips*: 0.00 ≤*X*^2^ ≤ 0.50, *p* = 0.480). Seventy-two percentage of the children with ASD emitted at least one spontaneous vocalization before the stimuli appeared and 86% did during stimuli displays (*X*^2^= 0.50, *p* = 0.480). The children with ASD uttered more spontaneous vocalizations during the display of all except the jagged edges stimuli (4.17 ≤*X*^2^ ≤ 5.14, *p* ≥ 0.023) (jagged edges: *X*^2^ = 1.33, *p* = 0.248; **Figure 3C**).

### Results of Comparisons between NT and ASD Children

#### Preferences and Feelings

Few differences were observed between children with ASD and NT children. Significantly more NT children than children with ASD perceived the spiral stimulus positively (Fisher test: *n*_1_ = 14, *n*_2_ = 14, *p* = 0.022). Moreover, significantly more NT children than children with ASD associated a neutral feeling with the jagged edges (*p* = 0.029). No significant differences could be evidenced between the two groups of children concerning preferred (Fisher test: *n*_1_ = 14, *n*_2_ = 14, *p* ≥ 0.326) or disliked (*p* ≥ 0.385) stimuli.

#### Behavioral Reactions

Significantly more children with ASD than NT children raised their eyebrows during the jagged edges display (*p* = 0.033) and children with ASD vocalized approximately four times more than NT children whatever the stimulus displayed (*p* ≥ 0.001; **Figure 3C**). No other significant differences could be evidenced between the two groups (*postural changes*: *p* ≥ 0.222; *redirected activities*: *p* ≥ 0.077; *self-centered activities*: *p* ≥ 0.376, and *facial movements*: *p* ≥ 0.098).

## Discussion

Our aim was to evaluate the emotional responses, i.e., preferences and behavioral reactions to simple shapes of NT adults and children and of children with ADS. Our first experiment revealed that neurotypical adults tended to perceive looming stimuli negatively as an increase of negative feelings was associated with these stimuli although few behavioral responses were recorded. Our second experiment revealed significant differences between the emotional responses of neurotypical children and those of children with ASD. Neurotypical children perceived the spiral stimulus positively, i.e., a curvilinear shape, whereas children with ASD perceived the jagged edges stimulus positively, i.e., an angular shape. Although some of the behavioral reactions of neurotypical children and of children with ASD behavior were similar, children with ASD smiled and vocalized more than did neurotypical children during displays. Finally, despite the wide age range of child participants (4–15 years), we found no age effect on the behaviors expressed by children in the presence of the stimuli. This indicates a transversal effect of simple shapes on children’s emotional responses.

Our results for both neurotypical adults and children confirm previous reports showing that humans consider static curvilinear shapes as “good” and static angular shapes as “bad” (e.g., Aronoff et al., 1992; Larson et al., 2007; Watson et al., 2012; Chen et al., 2015). When a looming movement was associated with these shapes, neurotypical adults’ feelings appeared to be slightly modified. Indeed looming movements added to visual stimuli tended to induce more negative responses by neurotypical adults than static stimuli. Moreover, previous studies found that looming movements induced humans and animals to react negatively (Ball and Tronick, 1971; Burger and Gochfeld, 1981, Hassenstein and Hustert, 1999; Carlile et al., 2006). Combining simple level properties could modify humans’ emotional responses (e.g., colors and shapes; Chen et al., 2015). However, according to our results looming stimuli were mainly perceived as neutral by neurotypical adults. So at this step, it is difficult to draw clear conclusions on neurotypical adults’ movement perception due to small sample size (*n* = 5 visual stimuli).

For the first time, behavioral data have been added to analyses of this type of research, and this enabled us to reveal that all our participants did not behave similarly in the presence of simple shapes, i.e., neurotypical adults presented one type of behavioral response and children presented several types of behavioral responses. The only subtle behavioral modification (i.e., frowning during stimulus displays) by neurotypical adults expressed surprise. This low level of expressiveness could be explained by (1) either the absence of negative emotional effects of the stimulus type and looming movement (2) or, if any effect exists, it may “remain” at a cognitive level inhibiting ongoing emotion-expressing behavior. Emotion regulation, i.e., “the processes by which we influence which emotions we have, when we have them, and how we experience and express them,” may be involved (Gross, 1999). Humans have many emotion-regulatory response options that allow them to habituate to an event or a stimulus (Gross, 2002). For example, cognitive changes are not necessarily associated with behavioral reactions. Another explanation could be that they are linked to the experimental context (e.g., behavior was video-recorded). When another person was in the experimental room, subjects expressed significantly fewer activities (i.e., vocalizations, body, and hand movements; Guerin, 1989). However, the fact that the subject was familiar with the person present seems to reduce behavioral inhibition (Buck et al., 1992). This behavioral response is also related to a child’s cultural background (e.g., Ekman, 1972; Eibl-Eibesfeldt, 1979; Ekman and Oster, 1979), gender (e.g., Davis, 1995), and family demands long before his/her first birthday (e.g., Ball and Tronick, 1971; Malatesta and Haviland, 1982).

Several stimuli induced both neurotypical children and children with ASD to display surprise by frowning, as neurotypical adults did. Contrary to neurotypical adults, children also presented other behavioral responses following a stimulus display. First, the fact that no postural changes linked to stimulus display were observed indicated that our simple shape stimuli elicited neither avoidance nor physical attraction, contrary to previous reports (e.g., Ball and Tronick, 1971). Self-centred activities of both neurotypical children and children with ASD decreased and so did neurotypical children’s redirected activities. As self-contact is known to have a soothing effect (Durier et al., 2015), children may be reassuring themselves while waiting for a stimulus to appear. Both children with ASD and NT children reacted similarly in other stressful experimental situations (e.g., encountering an unfamiliar pet; Grandgeorge et al., 2011, 2012). This decrease of self-centred activities may also be linked to attention focusing (observed for threat-relevant stimuli; Hillstrom and Yantis, 1994; Franconeri and Simons, 2003). Interestingly, this decrease occurred when neurotypical children perceived angular shapes (i.e., jagged edges) and when children with ASD perceived curvilinear shapes with a social component (i.e., head character). Children with ASD smiled and vocalized more during stimulus displays. Smiling is mainly produced socially and has social implications but it also expresses happiness (Kraut and Johnston, 1979); this last interpretation may be privileged in relation to the social difficulties of children with ASD (American Psychiatric Association [APA], 1994; Filliter et al., 2015) and the experimental design. Nevertheless, we cannot exclude that smiling, without feeling happy, might lower subjects’ stress level, as previously suggested concerning an unpleasant task (neurotypical adults; Kraft and Pressman, 2012) or stressful situations for instance in the presence of an unknown pet (children with ASD and neurotypical children; Grandgeorge et al., 2012) when children with ASD uttered more vocalizations than did neurotypical children (Grandgeorge et al., 2012); this results agrees with our present observations. As children with ASD and neurotypical children use these different behaviors to regulate their emotional responses, we hypothesized that emotion regulation induced by simple shapes differed between children and adults and that the expression of emotion regulation can depend on whether the child is neurotypical or has ASD.

Hence, the emotional responses induced by simple shapes are not universal (e.g., cultural background; Ekman, 1972; Eibl-Eibesfeldt, 1979; Ekman and Oster, 1979). They are modified by atypical development. For example, Rosenbloom (2006) highlighted correlations between color preferences and psychiatric disorders. Here, we evidenced differences between children with ASD and neurotypical children for both their preferences and behavioral responses. On the one hand, neurotypical children perceived the spiral stimulus positively (i.e., a curvilinear shape) whereas children with ASD perceived the jagged edges stimulus positively (i.e., an angular shape). On the other hand, children with ASD smiled and vocalized more than did neurotypical children during stimuli displays. These differences can be explained in several non-exclusive ways. At a neural level, Larson et al. (2009) showed that simple threat-relevant stimuli (i.e., downward V shape) recruit a neural circuitry implicated in affect and affective perception (e.g., amygdala, insula, subgenual anterior cingulate cortex). However, we do not know whether all simple shape stimuli recruit the same neural circuitry. Many reports (e.g., Barnea-Goraly et al., 2004; Thakkar et al., 2008; Uddin and Menon, 2009; Libero et al., 2014; Wegiel et al., 2014) show that these brain regions differ either in function, size or circuitry between neurotypical and people with ASD. These characteristics of brains of people with ASD are related to social and communication impairments observed related to ASD, especially to emotions (Monk, 2008). Mazefsky and White (2014) proposed that emotion regulation of people with ASD could be inherently disrupted so as to elicit aberrant behavior in highly emotional situations (Konstantareas and Stewart, 2006). This may explain why subtle emotional situations (i.e., looking at simple shapes) induced particular behavioral responses and simple shape preferences. Last, but not least, a possible explanation involves sensory alterations leading people with ASD to be attracted to, or repulsed by, visual stimuli (Ben-Sasson et al., 2009). Although the preferences of people with ASD and of neurotypical people seem to be linked to simple level properties of stimuli such as movement (Gepner et al., 2010), color (for a review; Simmons et al., 2009), or shape (as shown here).

Thus, to our knowledge, our study is the first to use ethological methods (Altmann, 1974) to record, analyse and evaluate effects of simple stimuli on emotional responses. Our observations yielded behavioral information concerning the underlying mechanisms involved and were complemented by questionnaire data on attitudes, feelings, and representations and neurophysiological investigations, i.e., information processing in the brain. All these information levels are important and interconnected as shown by Bush et al. (1989) and by Gross (2002).

Our study has limits because we used different experimental designs for children and for adults because preliminary experiments showed that the first experiment was too long for children with ASD. Moreover, not all children in the clinical sample met criteria for ASD on the ADI-R. Thus, further studies should repeat this experiment with another sample of children with ASD reducing the number of stimuli but retaining the two conditions, i.e., static versus looming. These data should help understand better the development of both perception of, and response to, simple-shape stimuli and movement effects considering that, for example, neurotypical children are less sensitive to visual looming than neurotypical adults in natural contexts (i.e., speeding vehicles when children try to cross the road; Wann et al., 2011).

Moreover, future studies should include more participants in order to study potential confusing effects of visual stimuli (e.g., stimulus complexity and familiarity). We are aware that spiral and jagged edges stimuli could have illusory properties. To our knowledge we do not know whether children with ASD are able to see illusory movements (Dakin and Frith, 2005), although NT children could. However, here, these two visual stimuli were presented with a looming movement so this movement probably captures their attention transcending the illusory properties of the stimuli.

Finally, Pierce et al.’s (2016) recent study using eye tracking revealed that children with ASD are more interested in dynamic geometrical shapes compared to social stimuli than neurotypical children. So eye tracking is a very useful and relevant tool to estimate interest and arousal of subjects and should be included in future studies.

## Conclusion

This study revealed, for the first time, that simple shapes induced specific emotional responses by humans, both adults and children, either with neurotypical development or ASD, but that their emotional responses, preference, and behavioral reactions differed according to development. Further studies should (1) involve subjects with other atypical developments and stimuli with other simple level properties (e.g., shape, symmetry, color; Reber et al., 2004) that are known to affect humans’ preferences, judgments, and decisions (Palmer et al., 2013) as well as (2) combine several methods to yield more detailed evaluations. We suggest that associating questionnaires, behavioral and neurophysiological investigations will be fruitful especially for adults who display few behavioral responses in the presence of simple shape stimuli (e.g., heart rate, skin conductance). Studying perception, recognition and response in the presence of relevant stimuli, beyond simple level properties, is crucial to fully understand the mechanisms and processes involved in human development and more widely, in individual survival.

## Ethics Statement

This study was carried out in accordance with the recommendations of “Comité de Protection des Personnes” with written informed consent from all subjects. All adult subjects gave written informed consent in accordance with the Declaration of Helsinki, and parents gave an informed consent to allow their child to participate in the study. The protocol waxs approved by the “Comité de Protection des Personnes, Ouest V.”

## Author Contributions

LH and MH conceptualized the study, chose the theoretical framework and chose the measures. LB, MD, and MG collected the data and wrote the “Materials and Methods” and “Results.” Then LB and MG wrote the paper together and, LH and MH read and revised the manuscript several.

## Conflict of Interest Statement

The authors declare that the research was conducted in the absence of any commercial or financial relationships that could be construed as a potential conflict of interest.
